# Supplementary figures and images for: DYF-4 regulates patched-related/DAF-6-mediated sensory compartment formation in C. elegans
Source: PLoS Genet. 2021 Jun 11;17(6):e1009618. doi: 10.1371/journal.pgen.1009618 (PMC8221789; doi:10.1371/journal.pgen.1009618)

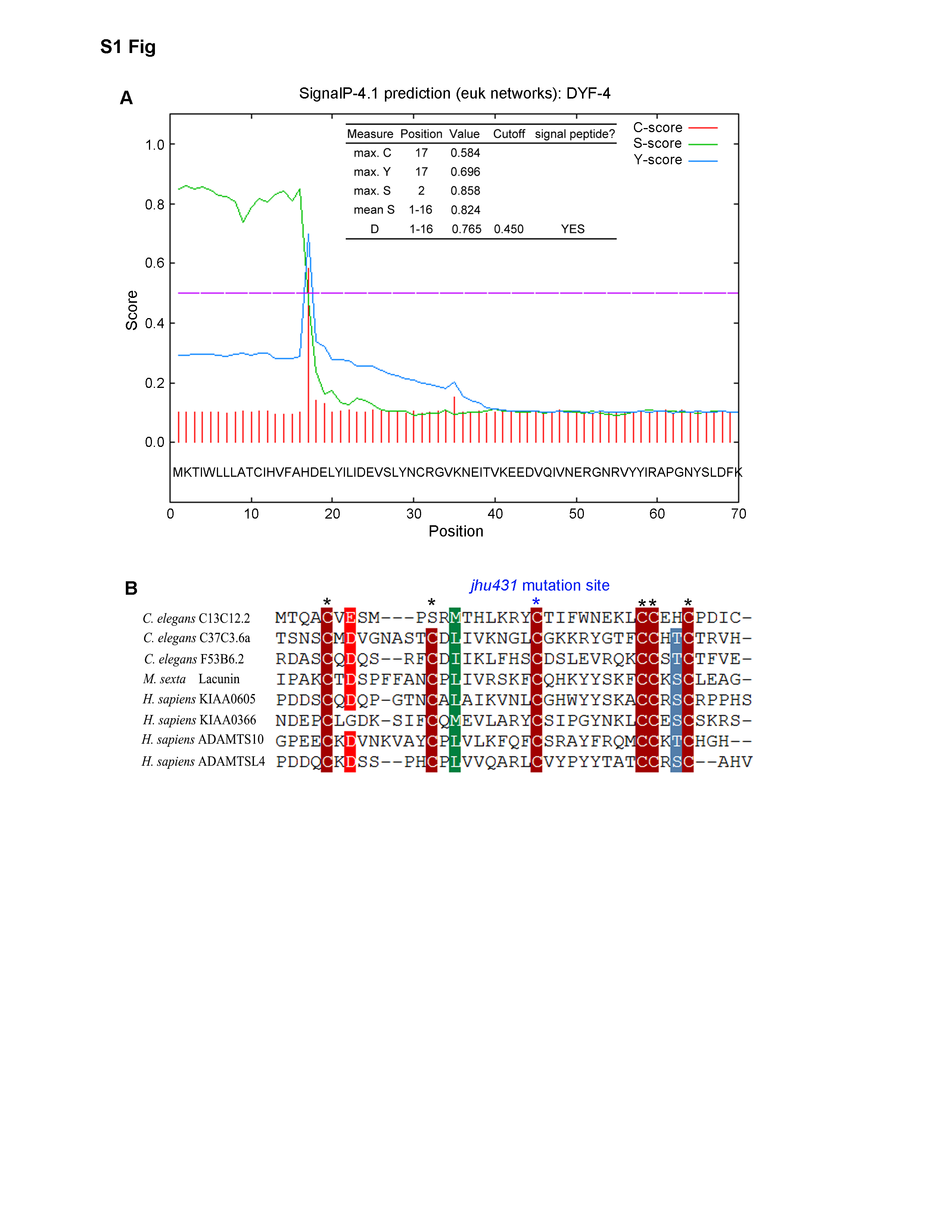

Supplement: S1 Fig — (A) Signal peptide prediction for DYF-4 by SignalP4.1. (B) Sequence alignment of the PLAC domain. *, the conserved cysteines in the PLAC domain. (TIF) [file pgen.1009618.s001.tif]

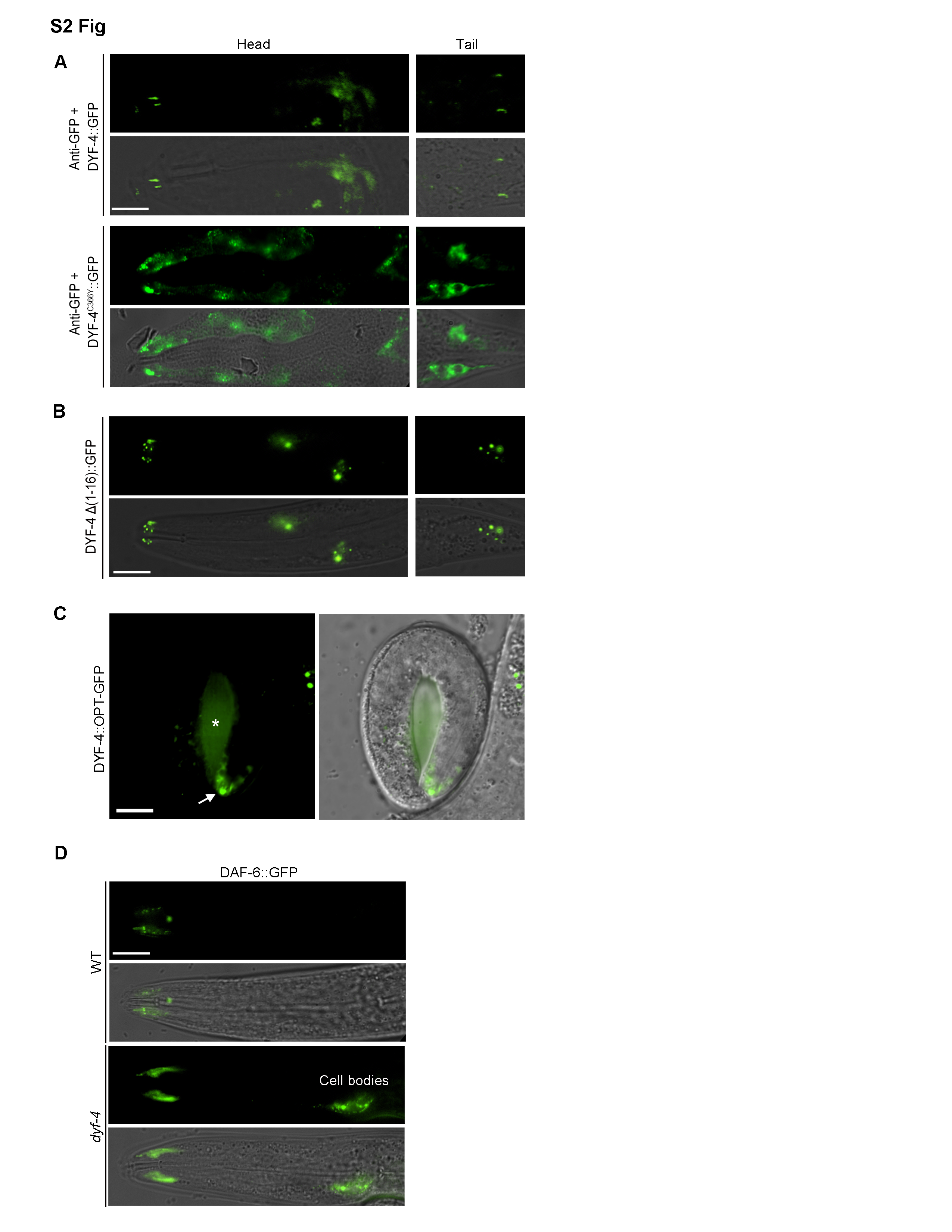

Supplement: S2 Fig — (A) The subcellular localization pattern of DYF-4C366Y::GFP stained by anti-GFP. Scale bar: 5 μm. (B) The subcellular localization pattern of DYF-4 Δ(1–16)::GFP. Scale bar: 5 μm. (C) The localization of DYF-4::OPT-GFP in a 3 fold embryo. White arrow indicates the localization of DYF-4 in amphid glial cells. White asterisk indicates the localization of DYF-4 between the embryo and the eggshell. (D) Subcellular localization of DAF-6::GFP in young adults of WT and dyf-4 worms. (TIF) [file pgen.1009618.s002.tif]

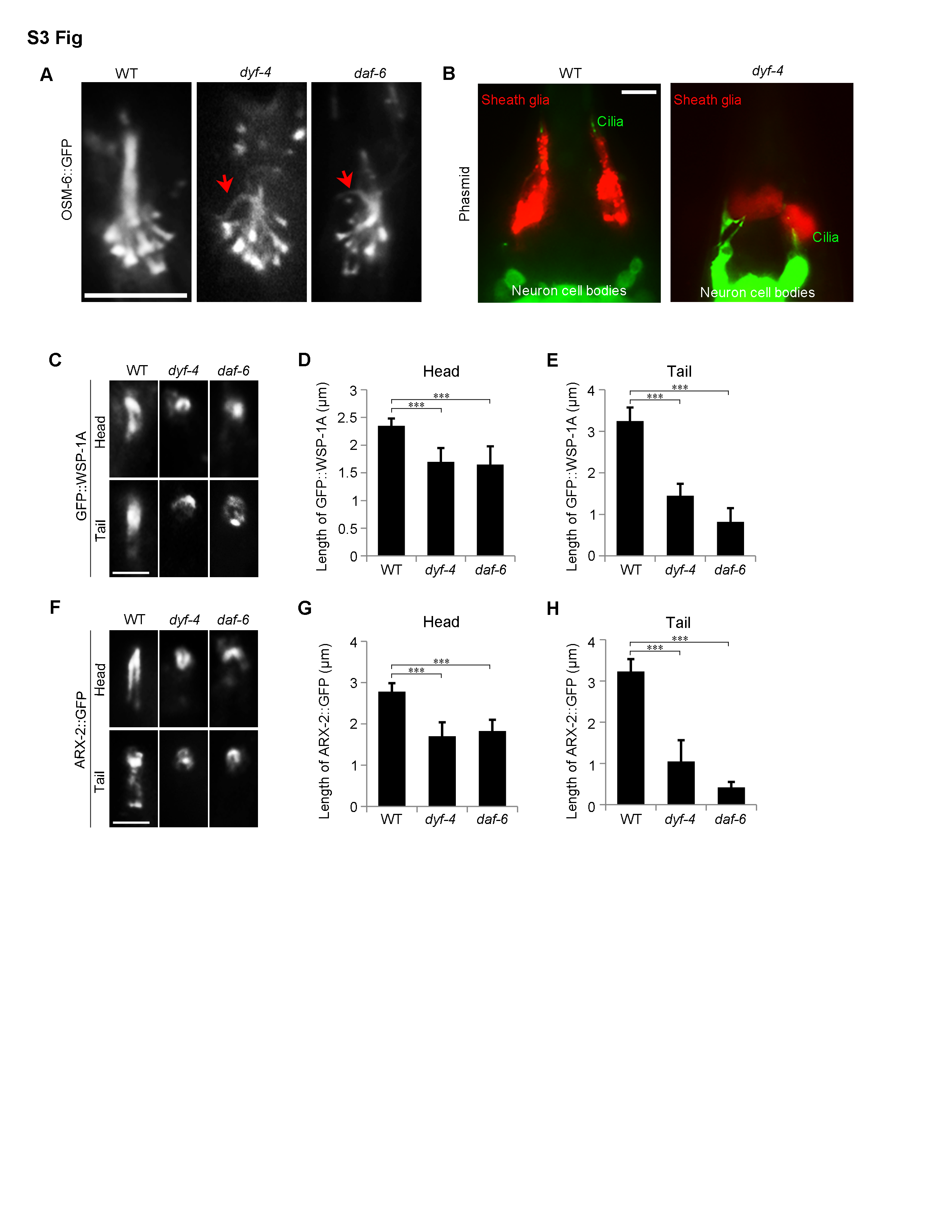

Supplement: S3 Fig — (A) Fluorescence micrographs of curved cilia in dyf-4 and daf-6 mutant worms. Curved cilia are indicated by red arrowheads. Scale bar = 5 μm. (B) Phasmids of WT and dyf-4 worms expressing F16F9.3pro::mCherry (Red, sheath glia) and OSM-6::GFP (Green, neurons). Sheath glia cells in dyf-4 mutants could not extend as in WT. Scale bar = 10 μm. (C) WSP-1A localization at the head and tail in WT, dyf-4(m158) and daf-6(e1377) worms. Scale bars: 2 μm. (D) Quantification of GFP::WSP-1A signal length in the amphids of WT, dyf-4(m158) and daf-6(e1377) worms. (E) Quantification of GFP::WSP-1A signal length in the phasmids of WT, dyf-4(m158) and daf-6(e1377) worms. (F) ARX-2 localization at the head and tail in WT, dyf-4 and daf-6(e1377) worms. Scale bars: 2 μm. (G) Quantification of ARX-2::GFP signal length in the amphids of WT, dyf-4(m158) and daf-6(e1377) worms. (H) Quantification of ARX-2::GFP signal length in the phasmids of WT, dyf-4(m158) and daf-6(e1377) worms. All data are presented as the mean ± SEM (n ≥ 50 for each genotype). ***P < 0.001 (Mann-Whitney test). (TIF) [file pgen.1009618.s003.tif]

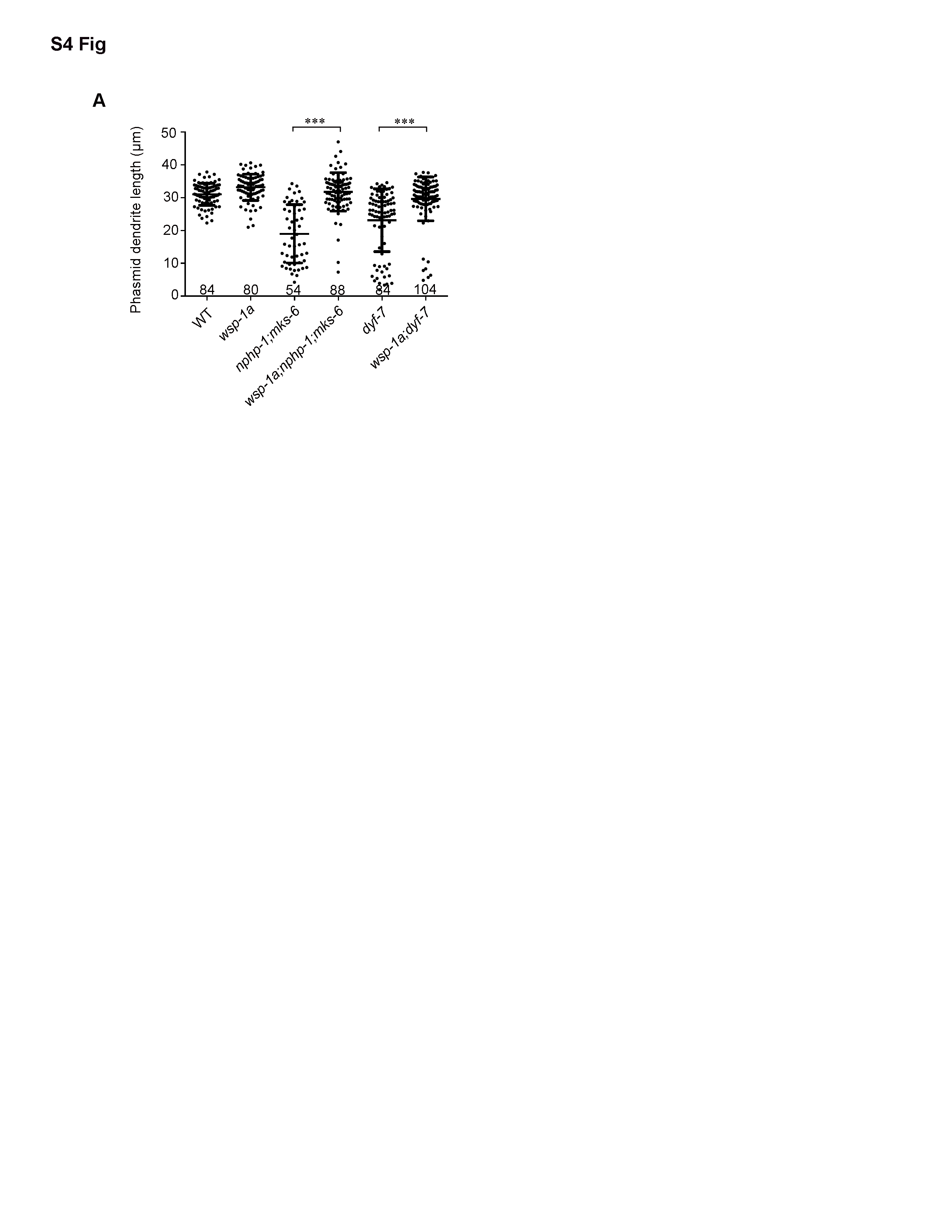

Supplement: S4 Fig — (A) Quantification of phasmid dendrite length in the indicated worm lines. The defective dendrite phenotype in dyf-7(ns117) mutants and nphp-1(ok500); mks-6(gk674) double mutants was rescued by wsp-1a(gm324). Each data point represents a single measurement. n represents number of dendrites analyzed. ***P≤0.001 (Mann-Whitney test). (TIF) [file pgen.1009618.s004.tif]

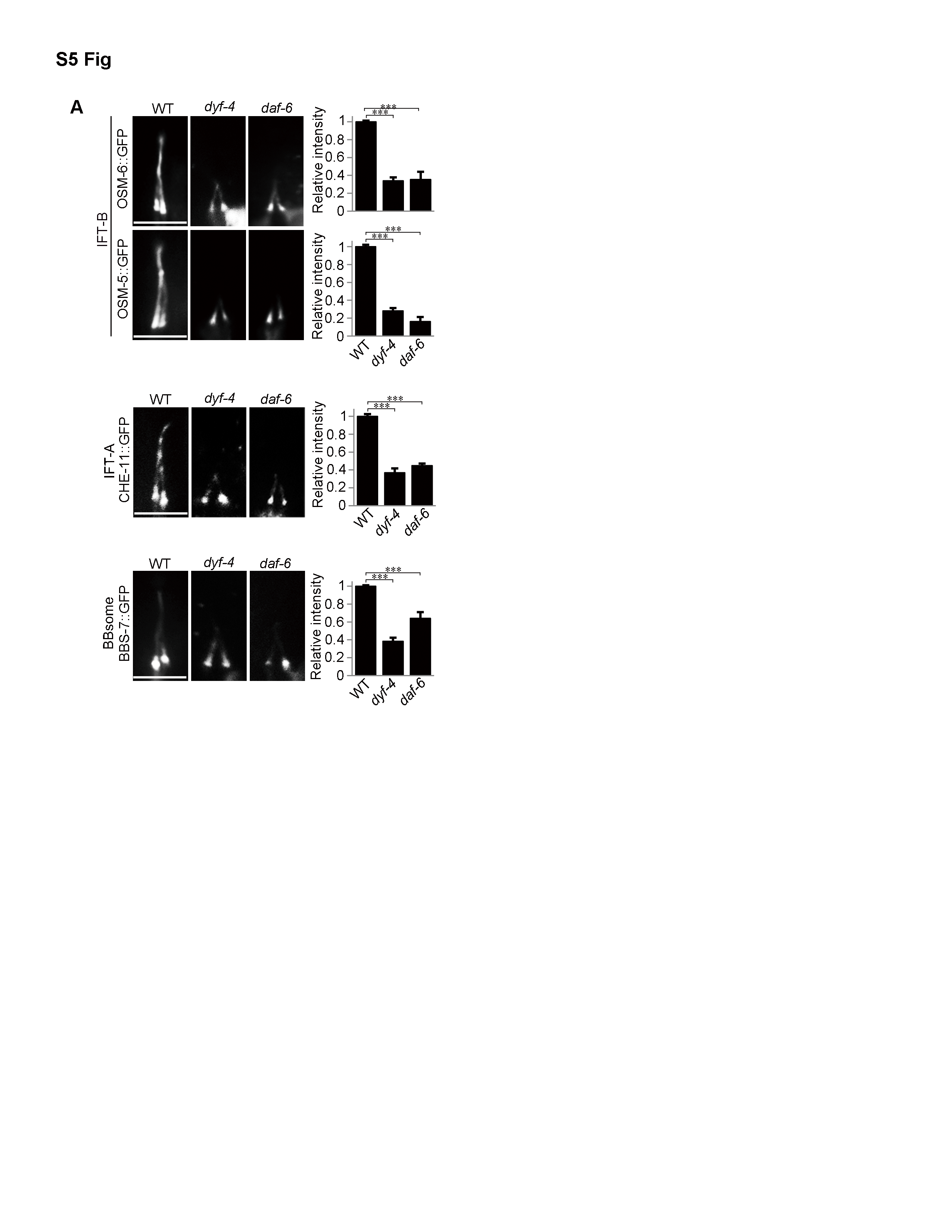

Supplement: S5 Fig — (A) Fluorescent micrographs and quantification of the relative fluorescence intensities in the phasmid cilia of WT, dyf-4(m158) and daf-6(e1377) worms expressing various IFT markers: IFT-B components OSM-6::GFP and OSM-5::GFP, IFT-A component CHE-11::GFP and BBsome component BBS-7::GFP. Data are presented as the mean ± SEM (n ≥ 60 for each genotype). ***P≤0.001 (Mann-Whitney test). Scale bars: 5 μm. (TIF) [file pgen.1009618.s005.tif]

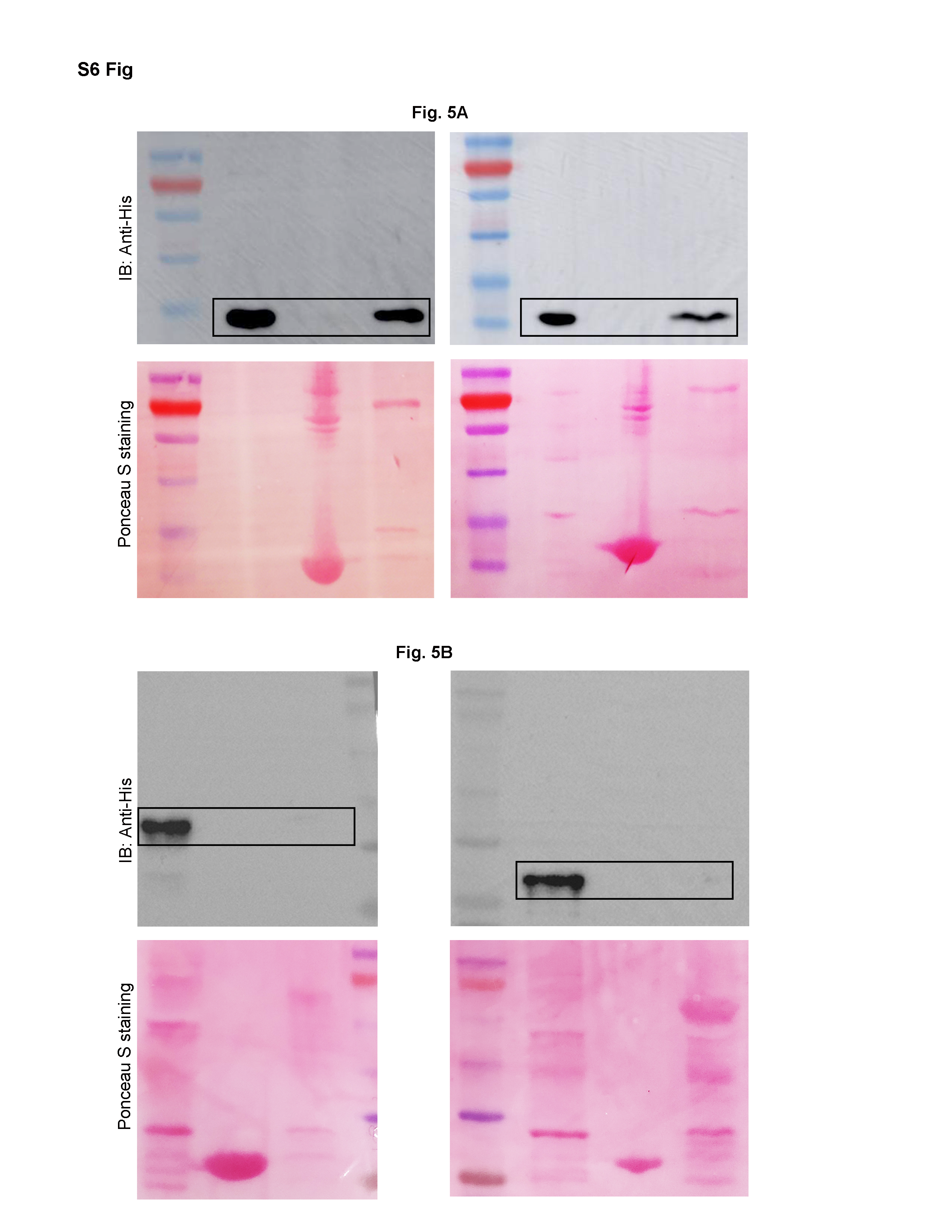

Supplement: S6 Fig — (TIF) [file pgen.1009618.s006.tif]
