# Supplementary material for: DYF-4 regulates patched-related/DAF-6-mediated sensory compartment formation in C. elegans
Source: PLoS Genet. 2021 Jun 11;17(6):e1009618. doi: 10.1371/journal.pgen.1009618 (PMC8221789; doi:10.1371/journal.pgen.1009618)
Supplement: S1 Table — (DOCX) [file pgen.1009618.s007.docx]

| **S1 Table. Worm strains used in this study.** | |
| --- | --- |
| **Strain name** | **Genotype** |
| QWL800 | *dyf-4 (jhu431) V; mnIs17 [osm-6::GFP] V* |
| SP1237 | *dyf-4 (m158) V* |
| QWL801 | *dyf-4 (m158) V; mnIs17 [osm-6::GFP] V* |
| CB1377 | *daf-6 (e1377) X* |
| QWL802 | *daf-6 (jhu500) X; mnIs17 [osm-6::GFP] V* |
| QWL803 | *daf-6 (e1377) X; mnIs17 [osm-6::GFP] V* |
| SP2101 | *mnIs17 [OSM6::GFP]V* |
| QWL804 | *qwaEx804 [Pdyf-4::GFP+pRF4]* |
| QWL805 | *qwaEx805 [Pdyf-4::DYF-4::GFP+pRF4]* |
| QWL806 | *qwaEx806 [Pdyf-4:: DYF-4^C366Y^::GFP+pRF4]* |
| QWL807 | *qwaEx807 [Pdyf-4:: DYF-4^Δ(1-16)^::GFP+pRF4]* |
| QWL808 | *qwaEx808 [Pdaf-6:: DYF-4::GFP+pRF4]* |
| QWL809 | *qwaEx809 [Pdyf-7:: DYF-4::GFP+pRF4]* |
| QWL810 | *qwaEx810 [Pdaf-6::DAF-6::GFP+pRF4]* |
| QWL811 | *qwaEx811 [Pdaf-6:: DAF-6::GFP+OSM-6::Mcherry+pRF4]* |
| QWL812 | *qwaEx812 [Pdyf-4:: DYF-4::GFP+OSM-6::Mcherry+pRF4]* |
| QWL813 | *qwaEx813 [Pdyf-4:: DYF-4::GFP+Pdaf-6:: DAF-6::Mcherry+pRF4]* |
| QWL814 | *dyf-4 (jhu431) V; qwaEx805 [Pdyf-4:: DYF-4::GFP+pRF4]; mnIs17 [OSM6::GFP] V* |
| QWL815 | *dyf-4 (m158) V; qwaEx805 [Pdyf-4:: DYF-4::GFP+pRF4]; mnIs17 [OSM6::GFP] V* |
| QWL816 | *dyf-4 (m158) V; qwaEx810 [Pdaf-6:: DAF-6::GFP+pRF4]* |
| QWL817 | *dyf-4 (m158) V; qwaEx806 [Pdyf-4:: DYF-4 ^C366Y^::GFP+pRF4]; mnIs17 [OSM6::GFP] V* |
| QWL818 | *daf-6 (jhu500) X; qwaEx810 [Pdaf-6:: DAF-6::GFP+pRF4]; mnIs17 [OSM6::GFP] V* |
| QWL819 | *daf-6 (e1377) X; qwaEx810 [Pdaf-6:: DAF-6::GFP+pRF4]; mnIs17 [OSM6::GFP] V* |
| QWL820 | *daf-6 (e1377) X; qwaEx805 [Pdyf-4:: DYF-4::GFP+pRF4]* |
| NG324 | *wsp-1a(gm324) IV* |
| QWL821 | *wsp-1a(gm324) IV; mnIs17 [OSM6::GFP] V* |
| EU603 | *lit-1(or131) III; him-8(e1489) IV* |
| QWL822 | *lit-1(or131) III; mnIs17 [OSM6::GFP] V* |
| VC20122 | *igdb-2(gk214668) IV* |
| QWL823 | *igdb-2(gk214668) IV; mnIs17 [OSM6::GFP] V* |
| QWL824 | *dyf-4 (m158) V; wsp-1a(gm324) IV; mnIs17 [OSM6::GFP] V* |
| QWL825 | *dyf-4 (m158) V; lit-1(or131) III; mnIs17 [OSM6::GFP] V* |
| QWL826 | *dyf-4 (m158) V; igdb-2(gk214668) IV; mnIs17 [OSM6::GFP] V* |
| QWL827 | *daf-6 (e1377) X; wsp-1a(gm324) IV; mnIs17 [OSM6::GFP] V* |
| QWL828 | *daf-6 (e1377) X; lit-1(or131) III; mnIs17 [OSM6::GFP] V* |
| QWL829 | *daf-6 (e1377) X; igdb-2(gk214668) IV; mnIs17 [OSM6::GFP]V* |
| QWL830 | *dyf-4 (m158) V; daf-6 (e1377) X; wsp-1a(gm324) IV; mnIs17 [OSM6::GFP]V* |
| QWL831 | *dyf-4 (m158) V; daf-6 (e1377) X; lit-1(or131) III; mnIs17 [OSM6::GFP]V* |
| QWL832 | *dyf-4 (m158) V; daf-6 (e1377) X; igdb-2(gk214668) IV; mnIs17 [OSM6::GFP]V* |
| QWL11 | *qwaEx11 [CHE-11::GFP+pRF4]* |
| QWL833 | *dyf-4 (m158) V; qwaEx11 [CHE-11::GFP +pRF4]* |
| QWL834 | *daf-6 (e1377) X; qwaEx11 [CHE-11::GFP +pRF4]* |
| QWL19 | *qwaEx19 [BBS-7::GFP+MKS-5::mCherry+pRF4]* |
| QWL835 | *dyf-4 (m158) V; qwaEx19 [BBS-7::GFP+MKS-5::mCherry+pRF4]* |
| QWL836 | *daf-6 (e1377) X; qwaEx19 [BBS-7::GFP+MKS-5::mCherry+pRF4]* |
| GOU2047 | *cas607[ARX‐2::GFP knock‐in] V* |
| GOU2049 | *cas723[GFP::WSP-1A knock‐in] IV* |
| QWL837 | *dyf-4 (m158) V; cas607[ARX‐2::GFP knock‐in] V* |
| QWL838 | *daf-6 (e1377) X; cas607[ARX‐2::GFP knock‐in] V* |
| QWL839 | *dyf-4 (m158) V; cas723 [GFP::WSP-1A knock‐in] IV* |
| QWL840 | *daf-6 (e1377) X; cas723 [GFP::WSP-1A knock‐in] IV* |
| QWL841 | *qwaEx841 [Pdyf-4::DYF-4::OPT-GFP+pRF4]* |
| QWL842 | *qwaEx842 [Pdyf-4::DYF-4::OPT-GFP+ OSM-6::Mcherry+pRF4]* |
| QWL843 | *daf-6; qwaEx841 [Pdyf-4::DYF-4::OPT-GFP+pRF4]* |
| QWL844 | *dyf-4 (m158) V; daf-6 (e1377) X* |
| QWL848 | *wsp-1a(gm324) IV; dyf-7(ns117)X* |
| QWL849 | *nphp-1* *(ok500) II; mks-6(gk674)I* |
| QWL852 | *wsp-1a(gm324) IV; nphp-1* *(ok500) II; mks-6(gk674)I* |
| QWL853 | *qwaEx853 [Pt02b11.3::GFP +Pgcy-5::mcherry + pRF4]* |
| QWL854 | *dyf-4; qwaEx853 [Pt02b11.3::GFP +Pgcy-5::mcherry + pRF4]* |
| QWL855 | *daf-6; qwaEx853 [Pt02b11.3::GFP +Pgcy-5::mcherry + pRF4]* |
| QWL856 | *mnIs17 [OSM6::GFP]V; qwaEx856 [Pf16f9.3:mCherry + pRF4]* |
| QWL857 | *dyf-4; mnIs17 [OSM6::GFP]V; qwaEx856 [Pf16f9.3:mCherry + pRF4]* |
