# Supplementary material for: DYF-4 regulates patched-related/DAF-6-mediated sensory compartment formation in C. elegans
Source: PLoS Genet. 2021 Jun 11;17(6):e1009618. doi: 10.1371/journal.pgen.1009618 (PMC8221789; doi:10.1371/journal.pgen.1009618)
Supplement: S2 Table — (DOCX) [file pgen.1009618.s008.docx]

**S2 Table. Peptide sequence information of extracellular loops in DAF-6 and CHE-14**

| **Name** | **Sequence** |
| --- | --- |
| DAF-6 ECL1 | RMTELRVDDPSYVFTPSDARWRREISVFNENWPLDENKFLPGKSFEAKRFVNILIRAKDGGSIMRDNVLHEIEILNQWIMNNISIPTDDLKFNLTYQDLCLSYDWVCGANEHIQMLLRRNDVNQILDLHFPRGGTKDTPVYLGGIFGDVQFFQNGTLSDAKLTQLFYFLKQDQKMVEEYSSKFSYALETFLNQVYSSDVITLSFAHYQSLEDGLDENAKA |
| DAF-6 ECL4 | REGLNPGNLVTNDHYIAKYFSDIKHFWRIGAQLHVAVLNPPNLTISENRNELLKVVSAFENTQYTLGREGTVFFLLEYLNYLSELNAEVEDTERLWKTKLNSWLKYTGGSTQWASNLKINKTDGSFQAFRFQIALKNFVEPNDHKHAAQLLRDIADHQPFNVVVYHEAFPFADQYLI |
| CHE-14 ECL1 | PIKLSQNAEIGFDTKDTDLSGPRLAWSRIQPSLMFSNRIAFSNPRPIDTDVQQPSAHPVSTKRNKRSWADNLLSAINQVACYDSPIPLMDHLSQFILEVPNYDAIFNLKFLDKLCQMQSNISKPLSRFDAFTPYRNIWSVANMFACISPNLRVNCTELDESDIKIVRKTIDNCWKYRTQIFECRDEKCQGQCATCRELPENCSSQIMYDLFYRLLPKQRDDTPFLVNTFLPVFTLTGYITQNIPVNVIIYDDLETSIIDYSKKNKFHLKGLLMDVKRDRLLAAALR |
| CHE-14 ECL4 | HLPEYNPLQLFTSDNLHEWYDNNAERNFEFVSAKIALPLTSRLVWGVEPIYSLSTFRANATSPLRSDPIFSLKTAGDVRKLARFLGTARQLPFVNHQPKFWPERFLDWSDKYPCARGFLCCNMTNPLFSDSYLDFCLRNSTSFLATSYNDTPIFDNKTFAFVGYTAMLPTSLKYNHRFKQLSKSFEMLEMTKPDNGWWAPEWWLMSTWFDLLSSIVQDCLS |
