# Supplementary material for: DYF-4 regulates patched-related/DAF-6-mediated sensory compartment formation in C. elegans
Source: PLoS Genet. 2021 Jun 11;17(6):e1009618. doi: 10.1371/journal.pgen.1009618 (PMC8221789; doi:10.1371/journal.pgen.1009618)
Supplement: Attachment — Submitted filename: Response to reviewers 0320.docx [file pgen.1009618.s009.docx]

**We would like to thank all Reviewers for taking the time and effort to review our manuscript. We sincerely appreciate all valuable comments and suggestions, which help us to improve the quality of our manuscript.**

**Reviewer #1:**

**Major comments:**

**Question 1.** The evidence for DYF-4 being an extracellular DAF-6 ligand is not very strong. Whilst the authors show that DYF-4 can bind two of the DAF-6 ECLs and demonstrate interdependent localisations, clearly more needs to be done to demonstrate a ligand-receptor relationship. Indeed, it is not clear from the data if DYF-4 is an extracellular protein, within the sensory compartment lumen, as the images do not provide enough resolution to demonstrate this. The findings of glial cell mislocalisation for DYF-4(delta 1-16) and DYF-4(C366Y) do not prove the extracellular properties of DYF-4. Also, it would be nice to show that the DAF-6/DYF-4 interaction from worm lysates. In fairness to the authors, they state in their discussion that more work needs to be performed to prove the extracellular ligand-receptor hypothesis. Therefore, they need to be more cautious about over interpretation. For example, in lines 218-219 of the results, and line 38 of abstract. I don’t expect the authors to do any more experiments for this comment, but just tone down the language in places and make it very clear what still needs to be done to prove the ligand-receptor model.

We agree with the reviewer that our current data are not sufficient to prove that DYF-4 is a ligand for DAF-6. The interaction between DYF-4 and DAF-6 is necessary but is not sufficient to prove that DYF-4 is the ligand of DAF-6. One of the key evidences to prove the ligand-receptor relationship is that the interaction between DYF-4 and DAF-6 can triggers the downstream signal of DAF-6. However, it is not clear what is the downstream signal of DAF-6 yet. Therefore, it is unlikely to prove that DYF4 is a ligand for DAF-6 at the current stage.

In fact, we know very little about the molecular function of DAF-6 in controlling the glia channel formation, we don’t even know if DAF-6 acts as a receptor like Patched. As far as we know, there is no evidence to support that DAF-6 is a receptor yet. Thanks to reviewer #2 for bring us attention to the working model of NPC1-NPC2. Another attractive possibility is that, like the relationship between NPC1 and NPC2, DAF-6 and DYF-4 cooperatively function in an intracellular vesicular compartment to regulate the sorting and recycling of certain cargos critical for vesicular dynamics and/or membrane formation. Understanding the detailed molecular function of DYF-4 depends on further detailed analyzing the molecular function of DAF-6 in future.

We really appreciate the reviewer for understanding our difficulties. As suggested, we revised the sentences in lines 38, 218-219, and toned down the description about ligand-receptor relationship.

**Question 2.** The paper’s major conclusion is that DYF-4 regulates sensory compartment formation, and this in turn leads to dendrite anchoring problems. Whilst the authors show mispositioning of amphid/phasmid cilia in dyf-4 L4-aged mutant, which by implication means the corresponding pores are abnormal, there is no actual data that shows dyf-4 pore lumen morphogenesis defects, as was presented previously for dyf-6 embryos/larvae by the Shaham group. Ideally, this new work would perform some TEM analysis of the dyf-4 amphid pore lumen at early ages to help prove the cause and effect relationship that is proposed. At the very least, they should include a statement in the discussion saying that TEM should be performed and results compared to dyf-6 worms.

To address this question, we visualized amphid sheath glia with GFP driven by the T02B11.3 promoter and the ASER neuron with mCherry driven by the *gcy-*5 promoter. The ASER cilium extends through sheath glia in WT, but it is bent and fails to extend through sheath glia in *dyf-4* mutants (Fig 4D), similar results were observed in *daf-6* mutants (*Oikonomou et al.*, PLoS Biol., 2011). We showed that phasmid sheath glia cells of *dyf4* mutants fails to extend as they did in WT (S3B Fig). To directly examine the glia channel defects, we performed TEM on the amphid channel of *dyf-4* adult mutants. As showed in Fig 4E, enlarged pore lumen and accumulation of granule vesicles were observed in *dyf-4* mutants. All these results indicate that the glia channel is indeed abnormal in *dyf-4* mutants.

We tried to use TEM to examine the amphid sensory compartment of *dyf-4* in embryo or early stages, but it was unsuccessful. Adult *C. elegans* is only 1 mm long, TEM of its amphid channel with right orientation is not easy. For samples in embryonic or early developmental stages, the challenge is even greater. On the other hand, experts are needed to identify sheath and socket cells in early developmental stage under EM. The detailed morphology abnormalities of sheath and socket glia during development in *dyf-4* mutant, as well as their comparisons with *daf-6*, need further investigation by experts in both TEM and glia cells in future. As suggested by the reviewer, we stated this in the discussion.

**Minor comments:**

1. Some quantification of the data in S3H should be provided, as was done in Fig. 1D.

Done.

2. Fig. S4A. What exactly does ‘severely collapsed’ mean. How was this determined for the analysis shown?

In Fig. S4A of origination version, we calculated the percentage of “severely collapsed dendrites” in amphids. All the amphid dendrites of *dyf-4* and *daf-6* mutants are slightly shorter, if cilia are located within half the distance from cell bodies to the nose tip, we define their dendrites as “severely collapsed”.

3. Line 361: I think the word ‘act’ is missing.

Revised. Thanks!

**Reviewer #2:**

**Specific comments**

1. Figure 1: Rather than a defect in “dendrite extension” (as the title states), this appears to be a defect in dendrite anchoring. Does the vertical line at left represent the nose tip position? If so, it appears amphid cell bodies have migrated to the appropriate location, but dendrite tips are not anchored at the nose. Similar comment applies to the phasmid defect. Actual dendrite length or the distance from nose tip to dendrite should be quantified here to capture this major aspect of the phenotype. To better understand the mutant phenotype, it would be helpful to visualize the socket and sheath glia positions and morphology (e.g. as in Low et al 2019).

Yes, the vertical line is the position of the nose tip. Exactly the same as what the reviewer said, the dendrite tips in *dyf-4* and *daf-6* mutants are not anchored where they should be as in WT.

Statistics of the distance between the ciliary base and the nose tip in amphid of *dyf-4* and *daf-6* mutants were shown in figure 1D and Figure 2D, respectively. Compared with WT, the basal bodies were significantly farther from the nose tip in mutants.

To better understand the mutant phenotype, we visualized amphid sheath glia with GFP driven by the T02B11.3 promoter and the ASER neuron with mCherry driven by the *gcy-*5 promoter. The ASER cilium extends through sheath glia in WT, but it is bent and fails to extend through sheath glia in *dyf-4* mutants (Fig 4D). We observed that phasmid sheath glia cells of *dyf4* mutants fails to extend as they did in WT (S3B Fig).We labeled amphid socked glia channel with GFP::WSP-1Aand ARX-2::GFP (Zhu et al., Cell Res., 2017), and found that the signal length of WSP-1 and ARX-2 was significantly shortened in *dyf-4*mutants compared to WT. We performed TEM on the amphid channel of *dyf-4* adult mutants, enlarged pore lumen and accumulation of granule vesicles were observed (Fig 4E). All these results indicate that glia channel morphogenesis is indeed abnormal in *dyf-4* mutants.

2. Figure1F: what is the “phasmid dendrite defect ratio”? It is not defined in the text or legend. Is it something different from the % of severely collapsed dendrites?

Generally, “phasmid dendrite defect ratio” refers to the percentage of dendrites with abnormalities, which could be slightly shorter or severely short. But in the case of *dyf-4* or *daf-6* phasmid, since all their dendrites are very short, the “phasmid dendrite defect ration” and “ % of severely collapsed dendrites” are actually the same.

3. Figure 2 and 2F: similar comments apply here as for Figure 1. Legend: “daf-6(e1377) was previously reported”. Please cite reference for this statement.

Done.

4. Figure 3: Photos are representative of how many animals imaged for these expts? What stage is being imaged? Is DYF-4 present in the developing glia of embryos (as implied in Figure 7)? Unfortunately, it is impossible to judge if DYF-4 is extracellular or in a vesicular compartment close to the apical membrane.

All worms are larvae or young adults. For Figure 3A, the images are representative of more than 30 worms from 3 independent experiments. For Figure 3C, since mCherry signal is weak and prone to photobleaching, the colocalization images are representative of more than 10 worms from 3 independent experiments.

DYF-4::GFP cannot be observed directly. We need to use indirect immunofluorescence method to enhance its signal. Due to the disadvantage of indirect immunofluorescence, such as high background, non-specific signals, it is difficult for us to use indirect immunofluorescence to conclude whether DYF-4::GFP is present in the developing glia of embryos.

However, during the revision, we replaced the GFP tag with OPT-GFP and found that DYF-4::OPT-GFP can be observed directly. In embryos, DYF-4::OPT-GFP could be observed (Please see the figure below). In larvae or young adults, DYF-4::OPT-GFP signals are localized in puncta/granule structures in amphids (S2E and F Fig), suggesting that they are in vesicular compartments. Interestingly, DYF-4::OPT-GFP signals tend to become a line, likely to be close to apical membrane. Of note, we could not exclude the possibility that partial DYF-4 may be secreted outside the cells.

5. Figure 4A,B: Photos are representative of how many animals imaged for these expts? What stage is being imaged? Given the eventual loss of the channel in daf-6 mutants, it may be more informative to look at an earlier stage of channel development, when it is still intact. The word “extracellular” appears within panel A erroneously.

As DYF-4::OPT-GFP signals can be directly observed, we replaced Figure 4A with new data. In the revised version, images for Figure 4A and B are representative of more than 100 worms.

In the original version, using indirect immunofluorescence methods, we showed that DYF-4::GFP signals were mislocalized and accumulated in amphids of *daf-6* mutants. However, using DYF-4::OPT-GFP, we found that the localization of DYF-4 is basically normal in *daf-6* mutants, only partial of amphids had abnormal DYF-4 signals (Fig 4A). Since non-specific signal is a common issue of indirect immunofluorescence, we think that our new results are more reliable. It is possible that, due to developmental abnormalities, amphids of *daf-6* mutants are more likely to have non-specific signals in indirect immunofluorescence staining. We apologize for this.

For DAF-6::GFP signal in *dyf-4* mutants in Figure 4B, we are confident that DAF-6 is mislocalized in either larvae or young adults of *dyf-4* mutants.

“extracellular” should be in panel C. Sorry for our mistake.

6. Figure 4C,D. What specific sequences are included in the ECL1 and ECL4 constructs? I do not see this information in the Methods section.

We did not find any predicted domains or motifs in ECL1 or ECL4 of DAF-6. The following is their sequence information:

ECL1 sequence:

RMTELRVDDPSYVFTPSDARWRREISVFNENWPLDENKFLPGKSFEAKRFVNILIRAKDGGSIMRDNVLHEIEILNQWIMNNISIPTDDLKFNLTYQDLCLSYDWVCGANEHIQMLLRRNDVNQILDLHFPRGGTKDTPVYLGGIFGDVQFFQNGTLSDAKLTQLFYFLKQDQKMVEEYSSKFSYALETFLNQVYSSDVITLSFAHYQSLEDGLDENAKA

ECL4 sequence:

REGLNPGNLVTNDHYIAKYFSDIKHFWRIGAQLHVAVLNPPNLTISENRNELLKVVSAFENTQYTLGREGTVFFLLEYLNYLSELNAEVEDTERLWKTKLNSWLKYTGGSTQWASNLKINKTDGSFQAFRFQIALKNFVEPNDHKHAAQLLRDIADHQPFNVVVYHEAFPFADQYLI

7. Figure 5: What igbd-2 allele was used here? It should be listed in legend.

*igdb-2 (gk214668)* was used. This allele has been described previously by *Wang et al.* (*Developmental biology 2017, 430:105-112*). The infromation has been listed in legend as suggested.

8. Figures 5, S4: How were “severely collapsed dendrites” defined?

For amphids, all the dendrites of *dyf-4* and *daf-*6 mutants are slightly shorter, if cilia are located within half the distance from cell bodies to the nose tip, we define their dendrites as “severely collapsed”.

For phasmids, if cilia are located at or very close the region of cell bodies, we define their dendrites as “severely collapsed”. In phasmids of *dyf-4* and *daf-6*, all cilia are located around the regions of cell bodies, all their dendrites are extremely short.

9. Figure 7 (model): The model should more clearly show what is being newly proposed based on the current work. Currently, this model is mostly summarizing data from prior Shaham or Heiman lab papers about daf-6 or dyf-7 (which should therefore be cited in the legend). There are no data shown in this paper to address whether the glial channel is initially open or whether socket and sheath glia detach in dyf-4 mutants. The placement of DYF-4 inside the sheath cell is particularly confusing, and it should be more clearly explained – are you proposing that DYF-4 is in a vesicular compartment (e.g. like NPC2) or in the extracellular matrix? Perhaps both models should be shown.

We appreciate the reviewer’s suggestions. We have redrawn the working model in the revision.

Currently, little is known about the molecular mechanism of Patched-related/DAF-6 regulating glia morphology. Based on published DAF-6 papers, it is generally believed that DAF-6 acts as a receptor like Patched. Therefore, in the original version, we thought that DAF-6 is a Patched related receptor. If DAF-6 is a receptor, DYF-4 should be in the extracellular matrix.

However, as far as we know, there is no any evidence to support that DAF-6 is a receptor yet. We really appreciate the reviewer for bring us attention to the NPC1-NPC2 working model. DAF-6 has previously been observed in punctate structures within the cytoplasm in vulva and excretory canal cells (*Perens et al., Dev. Cell, 2005*). We observed that DYF-4::OPT-GFP is localized in granular structures in amphids (S2E and F Fig). Therefore, it is very possible that, like the relationship between NPC1 and NPC2, DAF-6 and DYF-4 cooperatively function in intracellular vesicular compartments to regulate the sorting and recycling of certain cargos critical for vesicular dynamics and/or membrane formation.

At current stage, both models are possible. Understanding the detailed molecular function of DYF-4 depends on further detailed analyzing the molecular function of DAF-6 in future.

10. It would be informative to mention NPC1/NPC2 precedent in the Discussion.

We discussed as suggested. We really appreciate the reviewer for bringing us attention to the NPC1/NPC2 working model.

11. Table S1: What is IsOSM-6? This is non-standard nomenclature. Also, Ex transgene names should have lab and numerical identifiers.

Revised. It is mnIs17 [*osm-6*::GFP]. We got it from CGC.

12. Statistical methods should be re-evaluated in consultation with a statistician. The authors use a student’s t-test for both categorical and continuous data. Categorical data (e.g. dyf or not dyf; severely collapsed or not) should be analyzed with a Fisher’s Exact test instead. A t-test is appropriate for continuous data (e.g. distance measurements), but only if the data have a normal distribution and similar variance between groups, which doesn’t always seem to be the case here. Consider a non-parametric test such as Mann-Whitney instead.

We thank the reviewer for this valuable suggestion. Revised as the reviewer suggested.

13. There are many typos or grammatical errors, some of which are listed below. A copy editor will be needed.

Thank you so much for correcting us. We revised the manuscript and have it proof-read by commercial language-editing service.

line 50: “how DAF-6 functions and be regulated”

Revised to “but the function and regulatory mechanism of DAF-6 are not fully understood”.

lines 99-100: glial channel formation requires the dendrite tip anchoring protein DYF-7 (Low et al., 2019), sheath cells fail to undergo extension in dyf-7 mutants (Heiman and Shaham, 2009).

Revised to “sheath cells cannot extend, and glial channels are disrupted in *dyf-7* mutants”.

Line 150: “defects described here is a new phenotype”

Revised to “defect described here is a new phenotype”

Line 180: “DYF-4 is at least partially colocalizes with DAF-6”

Revised.

Line 188: “DYF-4 function in a same pathway as DAF-6”

Revised.

Line 203: “is the results of”

Revised.

Lines 248-249: “results in severely dendrite elongation defects …”

Revised. Changed to “results in severe dendrite elongation defects …”

**Reviewer #3:**

**Question 1**. The authors interpret their results in terms of morphogenesis of the glial channel. However, the data mostly relate to dendrite length and the glial channel is not examined directly at all. This is a major shortcoming. The authors seem to use dendrite length as a proxy for glial channel morphogenesis but it is not clear what the glial channel defects are, when they appear, or how (if) they relate to dendrite/cilia development.

We thank the reviewer for this important criticism. We have now provided the following evidences to show the glial channel defects in *dyf-4* mutants:

1. we visualized the amphid sheath glia with GFP driven by the T02B11.3 promoter and the ASER neuron with mCherry driven by the *gcy-*5 promoter. The ASER cilium extends through sheath glia in WT, but it is bent and fails to extend through sheath glia in *dyf-4* mutants (Fig 4D), similar results were reported previously in *daf-6* mutants (*Oikonomou et al.*, PLoS Biol., 2011).

2. We showed that phasmid sheath glia cells of *dyf4* mutants fails to extend as they did in WT (S3B Fig).

3. We labeled amphid socked glia channel with GFP::WSP-1Aand ARX-2::GFP which localize along the socked glia channel (Zhu et al., Cell Res., 2017), and found that the signal length of WSP-1 and ARX-2 was significantly shortened in *dyf-4*mutants compared to WT (S3C-H Fig).

4. To directly examine the glia channel defects, we performed TEM on the amphid channel of *dyf-4* adult mutants. Indeed, enlarged pore lumen and accumulation of granule vesicles were observed in *dyf-4* mutants (Fig 4E).

All these results indicate that, *dyf-4* mutants have abnormal glial channels.

Previous EM studies of *daf-6* mutants have demonstrated that the initial stages of sheath and socket channel development are unperturbed, but sheath lumen starts bloating from embryo about 420 min post-fertilization (before cilia have formed) and gradually becomes more and more serious as it grows up. Starting from L2, the sheath and socket channels are no longer continuous, and dendrite endings become disorganized (Oikonomou et al., 2011; Albert et al., 1981). Since all the phenotypes of *dyf-4* mutants observed so far are similar to *daf-6* mutants, we believe that glia channel defects in early development of *dyf-4* mutants are also similar to those of *daf-6* mutants.

We tried to use TEM to examine the amphid sensory compartment of *dyf-4* in embryo or early stages, but it was unsuccessful. The detailed morphology abnormalities of sheath and socket glia during development in *dyf-4* mutants needs further investigation by experts in both TEM and glia cells in future.

**Question 2**. The authors interpret their results in terms of developmental defects. However, the data relate to L4 stage phenotypes. Embryos (or even L1s) are not examined. This is another major shortcoming, especially in light of the relationship to DAF-6, which has progressively more severe defects, becoming extremely disorgnized by L4/adult. daf-6 dendrites are short only in adults, not in L2 larvae (Albert et al. 1981). It is not clear if dyf-4 affects dendrite/cilia development directly or if these are secondary defects that develop later as the structure becomes increasingly disorganized, as is the case for daf-6.

To answer this question, we examined the dendrites in young *dyf-4* larvae. As showed in Figure below, in phasmids, at L1 stage, the dendrite length of *dyf-4* is comparable to that in WT, but at L2 stage, the dendrite length of *dyf-4* is significantly shorter than that of WT. Consistently, in amphids, at L1 stage, the distance between the cilia base and the nose tip in *dyf-4* mutants is similar to that in WT. These results suggested that, similar to *daf-6* mutants, dendrite defects in *dyf-4* mutants is developed later and likely to be a secondary effect.

**Question 3**. Many of the results are inconsistent with previously published work. In principle this is not an issue but there should be some attempt to acknowledge/reconcile the differences. The defining phenotype of daf-6 mutants is the accumulation of vacuoles/enlarged secretory structures at the distal glial channel which does not seem to be seen here. Conversely the authors report shortened cilia which is not consistent with previous studies. Can the authors account for these differences?

In fact, almost all of our results are consistent with previously studies.

As showed in Fig 4C, vacuolar structures were observed in *dyf-4* mutants.

Previous studies on DAF-6 did not focus on the cilia length, the only data about cilia is that IFT protein CHE-2 was able to target to cilia in *daf-6* mutants (Perens et al., Dev. Cell, 2005, *“To determine whether cilia components were misexpressed or mislocalized in daf-6(e1377) animals, we examined localization of the cilia resident protein CHE-2 (Fujiwara et al., 1999). Functional CHE-2::GFP protein was localized to amphid cilia in 20/20 daf-6(e1377) animals examined, suggesting that at least one cilia component was properly localized in these mutants.”**).* Our data is consistent with this piece of data, IFT components were able to target to cilia.

All the IFT markers we used consistently showed that cilia are shortened in both *dyf-4* and *daf-6*, we are very confident in this phenotype.

**Question 4**. The localization defects are interesting but are more consistent with a chaperone role in which DYF-4 and DAF-6 mutually require each other for trafficking to the cell surface. However the authors' interpretation is that DYF-4 is an extracellular ligand for DAF-6, acting in the glial channel, despite the fact that DYF-4 cannot rescue when expressed from the neurons. This is very confusing and seems to contradict their results.

We thank the reviewer for raising this question. In the revised version, we have toned down the description about ligand-receptor relationship.

Currently, little is known about the molecular mechanism of DAF-6 regulating glia morphology. Based on published DAF-6 works, it is generally believed that DAF-6 acts as a receptor like Patched. Therefore, in the original version, we thought that DAF-6 is a Patched related receptor. If DAF-6 is a receptor, DYF-4 may be a ligand/ regulator of DAF-6. Of course, DYF-4 may also be a chaperone for DAF-6. But there is no precedence for a secreted protein as a chaperone of SSD-protein.

As far as we know, there is no evidence to support that DAF-6 is a receptor yet. Thank the reviewer #2 for bring us attention to the NPC1-NPC2 working model. It is also possible that, like the relationship between NPC1 and NPC2, DAF-6 and DYF-4 cooperatively function in intracellular vesicular compartments to regulate glia morphology.

At current stage, both models are possible. Understanding the detailed molecular function of DYF-4 depends on further detailed analyzing the molecular function of DAF-6 in future.

**Question 5**. The genetic interaction experiments are also very confusing. dyf-4 and daf-6 do not interact genetically with dyf-7, but suppressors of dyf-4 and daf-6 suppress dyf-7. What is the significance of this? The authors conclude "there are genetic interaction between glia compartment morphogenesis, dendrite anchoring complex and the TZ in dendrite extension". But, it is not clear that any of these mutants are affecting the same steps – for example, dyf-7 dendrite extension defects occur in embryos, and daf-6 dendrite length defects do not occur until late larval development. These experiments also use a hypomorphic allele of dyf-7 which complicates the analysis. All of these suppressor/enhancer experiments seem quite preliminary and inconclusive.

For synergetic interaction assay between two mutants, alleles that are two sever or too light are not suitable for analysis. As the null allele of *dyf-7* is to severe, it is not suitable for genetic interaction analysis. Previously, it has been reported that TZ mutants genetically interact with a hypomorphic allele of *dyf-7* in dendrite anchoring (Schouteden et al., 2015).

The phasmid dendrites in *dyf-4* or *daf-6* single mutants collapsed completely, so they are not suitable for synergetic interaction assay. Interestingly, we observed that suppressors of *daf-6* partially suppress the defects in dendrite length in TZ mutants or *dyf-7* mutants, suggesting that, at least in phasmid, there are genetic interaction between glia compartment morphogenesis, dendrite anchoring complex and the TZ in dendrite extension.

In the context of amphid, the dendrites of *dyf-4* or *daf-6* mutants are only slightly shortened. Therefore, it is possible that amphid dendrites of *dyf-4* or *daf-6* mutants are not suitable for synergetic interaction analysis. In order to reduce the confusion, we deleted this part of the data.

DYF-7 anchors dendrite tips in the extracellular matrix of glial channels. Although DYF-7 anchoring complex forms in embryo, glia channel defects in later stage will definitely change the position or composition of extracellular matrix, which will in turn affect the position of dendrites. Extracellular matrix may be the linker of all these dendrite length related mutants.

**Question 6**. The GST pull-down experiments are potentially promising but also seem preliminary and to lack appropriate controls. It is curious that both the extracellular loops of DAF-6 independently bind DYF-4 equally well. Perhaps DYF-4 is just "sticky"? It would help to include some negative controls, for example extracellular loops of related proteins like CHE-14 that are not expected to bind DYF-4, or point mutations in the DAF-6 loops that have been shown to disrupt its function. S253P in loop 1 and F568L in loop 4, which disrupt localization of DAF-6 similarly to loss of dyf-4 (Perens et al. 2005), would be especially good candidates to investigate.

We thank the reviewer for raising this question. To address this question, we examined the interaction between DYF-4 and 2 extracellular loops of CHE-14, no interactions were detected (Please see the figure below), suggesting the interaction between DYF-4 and DAF-6 has specificity.

We thank the reviewer for bringing us attention to those point mutations of DAF-6. It will be interesting to test whether these mutations affect the interaction between DAF-6 and DYF-4 in future.

**Question 7**. The model figure seems to place WSP-1 and LIT-1 upstream of DAF-6 and DYF-4. This is not consistent with the genetic data, in which loss of these WSP-1/LIT-1 suppresses loss of DAF-6/DYF-4. The model figure itself adds very little to the paper other than re-stating the phenotypes, while omitting the key features of the daf-6 mutant (over-expansion of the sheath pore in the embryo).

The working model has been revised. Please see our new model in Fig 7.
