# Supplementary material for: DYF-4 regulates patched-related/DAF-6-mediated sensory compartment formation in C. elegans
Source: PLoS Genet. 2021 Jun 11;17(6):e1009618. doi: 10.1371/journal.pgen.1009618 (PMC8221789; doi:10.1371/journal.pgen.1009618)
Supplement: Attachment — Submitted filename: Response to reviewers 2nd.docx [file pgen.1009618.s010.docx]

**Reviewer #1:**

Authors have done a good job addressing my comments.

We are very grateful to the reviewer for his positive comments, and sincerely thank him for his time and effort on our manuscript.

**Reviewer #2:**

Hong et al provide compelling evidence that the secreted protein DYF-4 functions with the Patched-related protein DAF-6 to affect the morphology of C. elegans glial sensory channels and their cognate sensory neurons. The discovery of a DAF-6 partner is very significant given the widespread importance of Patched, Dispatched, NPC1, and other Patched-related proteins; therefore this paper should be of broad interest.

The revision now provides more direct evidence for glial channel defects by visualizing the sheath and socket glia with fluorescent markers and by TEM; this confirms that the dyf-4 and daf-6 phenotypes are really similar.

The authors have also removed claims for a ligand-receptor relationship between DYF-4 and DAF-6, and instead more appropriately consider several possible models for their relationship.

We sincerely thank the reviewer for his/her time and effort on our manuscript, and greatly appreciate his/her positive comments.

There are remaining issues:
There are many places where the authors provided clarifying information in the "response to reviewers", but then failed to include that information in the actual manuscript. This information is needed for readers to understand (and believe) results shown in the paper.

We thank the reviewer’s suggestion. We have now included that information in the revised version (Fig 2D, Fig 5B and S2C Fig).

1. Figure 1B, 2B legends should explain that the vertical line at left represents the nose tip position.

The sentence has been added to Figure legends. Thanks!

2. Figure1F, 5C, 6D: The “phasmid dendrite defect ratio” and/or "severely collapsed dendrites" should be defined in the Methods or legends.

Since in *dyf-4* and *daf-6* mutants, “phasmid dendrite defect ratio” and “severely collapsed dendrites” are actually the same. To be consistent, in the revised version, we used “severely collapsed dendrites” instead of “phasmid dendrite defect ratio” in Fig 1F and Fig 2F.

As suggested, “severely collapsed dendrites” has been defined in figure legends. “Dendrites with cilia located near the cell bodies are defined as severely collapsed dendrites.”

3. Regarding the timing of the phenotype, L1 and L2 data shown in the "response to reviewers" (Rev 3 Question 2) should be shown and discussed in the manuscript (could be supplemental).

We have put the data to Fig 2G in the revised version. Thanks!

4. The DYF-4::OPT-GFP embryo image included in the "response to reviewers" should be shown in Figure 3 or S2, as it clearly shows that the protein is present during embryonic development and is secreted, accumulating between the embryo and the eggshell.

Thanks! The data has been added to S2C Fig.

5. Figures 3 and 4: The legends should list the stage and say that these are "representative of more than 100 worms" (or whatever is the case).

Done.

6. Figure 3E. The specific sequences included in the ECL1 and ECL4 constructs should be listed in the Methods section or in a supplemental Figure/Table.

As suggested, sequences have been listed in S2 Table in the revised version.

7. Figure 3E. The control pull-down experiments done with CHE-14 (shown in response to reviewer 3) should also be shown in the manuscript (could be supplemental).

We have now included this data in Fig 5B.

8. Table S1: The OSM-6::GFP nomenclature was fixed, but a large number of QW strains still use non-standard nomenclature. All Ex transgenes should be named with lab and numerical identifiers.

Revised. Thank you so much for your correction!

9. There are still typos or grammatical errors, some of which are listed below.

Fig. S6: The figure panel itself says it refers to Fig. 4C, whereas the legend refers to Fig. 3E.

Revised.

Lines 146-148: "It is likely that the abnormal morphology of glial channels changes the anchoring position of the tips of dendrites, which indirectly leads to the shortening of dendrites". This sentence sounds like pure speculation - please add "see below" or give some indication that data about glial channel morphology will be shown later in the manuscript.

“(see data in Fig 4)” has been added to the sentence.

Line 190: "only partial of amphids"

Has been revised to “only a fraction of amphids”.

Line 202: "Dendrite defects are much more severed" (severe)

Revised. Thanks for correcting our mistake.

10. Some of the data in the Supplemental Figures are quite important and really should be shown in a main Figure instead.

We thank the reviewer’s suggestion. All these data have been incorporated into main figures in the revised version.

For example, rescue data in Fig. S2B (text lines 156-159) argue for a glia cell-autonomous requirement for DYF-4, which is more consistent with a role in DAF-6 trafficking or some other vesicular process, and less consistent with a ligand-receptor relationship on the external cell surface (as pointed out by Reviewer 3). The data could be highlighted and discussed in this context.

This data was shown in Fig 3C, and discussed in the discussion (text lines 335-337).

DYF-4::OPT-GFP imaging data in Fig. S2F shows the punctate localization that leads authors to propose DYF-4 might be in vesicles (text lines 177-178).

This data was shown in Fig 3G.

Double mutant data in Fig. S3I is critical for the conclusion that dyf-4 and daf-6 act in the same pathway.

The data was shown in Fig 5C.

**Reviewer #3:**

The authors have clearly made a diligent attempt to respond to the comments. The manuscript has been improved by their revisions. By being somewhat more cautious in its conclusions and providing better support for the central claims, the revised manuscript really highlights the strengths of this exciting story.

We greatly appreciate the reviewer’s positive comments, and sincerely thank the reviewer for his/her time and effort on our manuscript.

Regarding my previous major comments:
1. The EM and other additional data related to visualizing defects in the glial channel have improved the story.

Thanks!

2. Regarding timing of the phenotypes, the data that are shared privately in the reviewer response are excellent. In my view this result, showing that dendrite defects only appear at the L2 stage, is essential to interpreting the results. **It needs to be included in the manuscript.**

Done. We have put the data to Fig 2G in the revised version. Thanks!

3. These clarifications and additions are very helpful.

Thanks!

4. The revised discussion of the DYF-4-DAF-6 relationship is a major improvement.

Thanks!

5. The discussion of genetic interactions remains somewhat confusing, and possibly misleading. It is really important to clarify in the text that dendrite defects in dyf-7, TZ mutants, and daf-6/dyf-4 arise at different developmental stages, suggesting that any genetic interactions among them would be indirect.

Thanks for your advice. We have discussed in the revised version (text lines 256-259).

The authors respond to my comments, "Although DYF-7 anchoring complex forms in the embryo, glia channel defects in later stage will definitely change the position or composition of extracellular matrix which will in turn affect the position of dendrites." My understanding is that DYF-7 controls dendrite length by preventing rupture of the developing sheath-socket-hyp epithelium in the embryo. It is not clear to me how the ECM would affect dendrite anchoring after the epithelium has already ruptured. It seems like these genes are acting at different developmental stages, and probably through different mechanisms.

We agree with the reviewer’s opinion that DYF-4/DAF-6, DYF-7 and TZ proteins may act at different developmental stages, and probably through different mechanisms.

My opinion in previous rebuttal letter is based on the hypothesis that DYF-7 acts in extracellular matrixes to anchor dendritic tips (*Heiman and Shaham, 2009 Cell*). If DYF-7 controls dendrite length by preventing rupture of the developing sheath-socket-hyp epithelium in the embryo, that mean that the rupture of the connection between the sheath glia and socket glia will result in short dendrites. If this is the case, the short dendrites in *daf-6* mutants might be caused by the disassociation of the sheath glia and socket glia which has been observed previously (*Oikonomou et al., 2011 PLOS Biology*).

6. The additional figure shared privately in the rebuttal letter is very helpful and strengthens an important conclusion of the paper. Because it addresses an important point that many readers will consider, I would **strongly encourage** the authors to add it to the manuscript.

We thank the reviewer’s suggestion. We have now included these data in Fig 5B of the revised version.

I have a few other minor comments that might improve the manuscript, but these should be handled at the authors' discretion and are not an impediment to publication:

1. In the introduction, perhaps revise to "the distal portions of *most* cilia extend through a pore formed by the membrane of the socket cell" to clarify that this is not the case for some cilia (AWA, AWB, AWC, AFD).

Revised as suggested. Thank you so much for pointing this out!

2. Several minor typos – "compartmentation" for "compartmentalization", "severed" for "severe", "socked" for "socket", "Gila" for "Glia" in Fig 7.

We apologize for our mistakes. Revised.

3. It is surprising that DAF-6 ECL1 and ECL4 both bind DYF-4. It would help to note whether these sequences share any motifs (does the 12 TM structure of DAF-6 look like a duplication of a more ancestral 6 TM sequence, with ECL4 homologous to ECL1?). As noted, the additional CHE-14 control included in the rebuttal letter would really help to shore up the specificity of this result.

DAF-6 originated from the duplication of 6 TM sequence is a very interesting hypothesis. Currently, we do not have any evidence to support this hypothesis, and we could not identify any motif in ECL1 and ECL4 of DAF-6. But, the alignment of two ECL sequences shows a similarity of 34.8% (Similarity Matrix: PAM250, see below).

4. "the localization of DYF-4::OPT-GFP is basically normal, only partial of amphids had abnormal DYF-4 signals". I think this should read "only a fraction of amphids", and it would be very helpful to cite what proportion (even a ballpark estimate, e.g. "~5%", "<20%", etc.).

Thanks for your advice. Revised to “only a fraction (~%10) of amphids” (text line 190).

5. In Fig. 4B, a zoomed-out view would help – is DAF-6 mostly stuck in the cell body? I am confused why a 12-TM protein is giving a diffuse cytoplasmic signal. It looks like soluble GFP. Perhaps clarify in the text what we are looking at.

A zoomed-out view image of DAF-6::GFP in *dyf-4* mutants has been added to S2D Fig in the revised version. Compared to WT, in addition to the accumulation in amphid sensory compartment region, the GFP signal does accumulate in cell bodies and shows punctate signals in *dyf-4* mutants (S2D Fig). We are not sure where these GFP signals are located, one possibility is the accumulation of vesicles.

6. In Fig. 5, the difference between % Dye-filling and % severely collapsed is potentially confusing. For example, wsp-1a; dyf-4 appears to be almost fully rescued for dendrite collapse but nevertheless fails to dye-fill. I think this is because many dendrites are of intermediate length – it might help to clarify this in the text. On a related note, it would help to be consistent between Fig. 5A and 5C in whether you show the fraction that are normal or defective (so WT would be 100% or 0% in both plots).

There are two reasons for the dye filling defect in *wsp-1a; dyf-4* double mutants: one reason is that some dendrites in *wsp-1a; dyf-4* mutants are indeed of intermediate length; the other one is that WSP-1 is required for cilia morphogenesis (*Zhu et al., 2017 Cell Research*), cilia tend to curve in *wsp-1* mutants. Although the amphid neurons of *wsp-1* mutant could take up DiI, 55% of the phasmid neurons of *wsp-1* mutant failed to take up DiI.

In addition, to be consistent, we showed the defects in both plots in the revised version.

7. The claim that "the actin cytoskeleton is likely the major downstream functional site of DYF-4-DAF-6-regulated glial function" seems like it goes far beyond what the data support. There does not seem to be any evidence yet to distinguish whether WSP-1 acts downstream of DYF-4/DAF-6 rather than in a parallel pathway. For example, if dyf-4/daf-6 defects arise by excess vesicle fusion to the forming channel lumen, then wsp-1 mutants might suppress this defect by reducing vesicle delivery to the apical surface. This seems quite possible, and dyf-4/daf-6 would not affect actin in this case at all. I would be very careful here.

We agree with the reviewer. This sentence has been deleted in the revised version.

8. In the model drawn in Fig. 7, showing DAF-6 as an inhibitor of glia channel morphogenesis suggests that loss of DAF-6 should improve channel morphogenesis – but in fact, loss of DAF-6 disrupts morphogenesis. I understand what you are trying to convey, but something like "glial channel growth" or "expansion" might be better.

Thank you so much for your suggestion. We have changed “glia channel morphogenesis” to “glial channel growth” in the revised version.
